# The Impact of Trimethylamine N-Oxide on Atrial Fibrillation Presence in Patients with Cardiovascular Disease

**DOI:** 10.3390/jox15010028

**Published:** 2025-02-07

**Authors:** Cristian Marius Florea, Radu Ovidiu Rosu, Ioan Alexandru Minciuna, Gabriel Cismaru, Dana Pop, Ana-Maria Vlase, Iuliana Nenu, Gabriela Adriana Filip

**Affiliations:** 1 Department of Physiology, Iuliu Hatieganu University of Medicine and Pharmacy, 400006 Cluj-Napoca, Romania; florea.cristian@umfcluj.ro (C.M.F.); iuliana.nenu@gmail.com (I.N.); adrianafilip33@yahoo.com (G.A.F.); 2 Fifth Department of Internal Medicine, Cardiology Clinic, Iuliu Hatieganu University of Medicine and Pharmacy, 400347 Cluj-Napoca, Romania; iaminciuna@gmail.com (I.A.M.); gabi_cismaru@yahoo.com (G.C.); pop67dana@gmail.com (D.P.); 3 Department of Pharmaceutical Botany, Iuliu Hațieganu University of Medicine and Pharmacy, 400337 Cluj-Napoca, Romania; gheldiu.ana@umfcluj.ro

**Keywords:** atrial fibrillation, TMAO, cardiovascular disease, atherosclerosis, risk factors, gut microbiota

## Abstract

**Background**: Atrial fibrillation is the most common human heart rhythm disorder, yet its underlying causes remain largely unknown. Trimethylamine N-oxide (TMAO), a by-product derived from the gut microbiota contributed by red meat, has been linked to numerous cardiovascular and metabolic diseases. **Aims**: This study aimed to evaluate the impact of serum TMAO levels on the occurrence of atrial fibrillation in patients with cardiovascular disease. **Results**: Utilizing a cross-sectional study design, fasting serum TMAO levels were measured and compared between 153 patients without cardiovascular disease and patients hospitalized for cardiovascular disease, stratified by the presence or absence of atrial fibrillation. While patients with more comorbidities had higher TMAO overall, the TMAO levels were not significantly different between cardiovascular disease patients with and without atrial fibrillation (*p* = 0.57). Moreover, there was no difference between atrial fibrillation progression phenotypes (*p* = 0.27). In multivariate analysis, a significant association was found with atherosclerotic cardiovascular disease (*p* = 0.04) and chronic kidney disease (*p* < 0.001), but there was no significant association between TMAO and atrial fibrillation (*p* = 0.9). **Conclusions**: Serum TMAO levels are not associated with the occurrence of atrial fibrillation and disease progression phenotypes in patients with cardiovascular disease, but are associated with ASCVD and CKD.

## 1. Introduction

Atrial fibrillation (AF) is the most prevalent clinical arrhythmia, with important morbidity and mortality and exerting a great pressure on healthcare expenditure worldwide [1]. AF onset and progression are related to numerous cardiovascular risk factors such as arterial hypertension (HTN), type 2 Diabetes Mellitus (T2DM), obesity, dyslipidemia and physical inactivity. The last edition of the European Society Guidelines for the management of AF emphasizes the importance of controlling and managing the burden of risk factors, which can significantly alter the natural history of AF by preventing or reversing adverse structural and electrical changes in the atrium [2], thus reducing the severity and recurrence of AF symptoms. Regardless, AF is a very complex arrhythmia and its pathogenesis is still largely unknown, with traditional risk factors only partially explaining its occurrence. Over the past decade, a plethora of evidence has shown that the gut microbiota can have a significant influence on human health through biologically active molecules [3]. Alterations in the composition of the gut microbiota have been associated with cardiovascular disease and traditional risk factors that influence the incidence and progression of AF, making the investigation of this interaction an attractive topic. In fact, studies have shown that specific alterations in the composition of the intestinal microbiota are associated with the incidence [4] and progression of AF [5,6]. Trimethylamine (TMA) is one of the most studied metabolites, derived exclusively from the metabolism of the microbiota of quaternary amines such as choline, carnitine, and lecithin, contained in great amounts in foods such as red meat, eggs and full-fat dairy products [7], related to a Western-type diet. TMA is further absorbed and oxidized in the liver by the flavin-monooxygenase family of enzymes to Trimethylamine-N-oxide (TMAO), which has been linked to numerous cardiovascular [8] and metabolic diseases [9,10]. In addition, TMAO has been associated with various cardiac and vascular functional and structural alterations that can promote AF, such as cardiac fibrosis and inflammation [11], endothelial dysfunction [12], platelet hyperreactivity and thrombosis [13], and even atrial electrophysiologic changes [14]. Although the relationship between AF and TMAO and vascular and cardiac tissue inflammation is quite clear, few studies have evaluated the association with systemic inflammatory cytokines in a clinical setting. Some studies have investigated the effect of TMAO on clinical AF, with the majority showing increased incidence [15] and progression [16] with higher levels of TMAO, but also negative studies showing no association [17]. In our previous studies, the proinflammatory effect of TMAO on the vascular system and tissues was noticed in a murine model, with mice supplemented daily for 3 months with different doses of TMAO. Additionally, increased serum inflammatory markers, oxidative stress and a higher expression of NF-κB, NRF2, SOD1 and iNOS in aortic rings were found [18,19]. Therefore, we hypothesized that there could be also a link between TMAO levels, AF incidence, and systemic inflammation in human patients.

In the present study, we aimed to provide insights into the potential links between gut microbiota-derived metabolites and cardiac arrhythmias by identifying the patterns or associations that may exist between TMAO and AF incidence. Specifically, we sought to determine the correlation between serum TMAO levels and the occurrence of atrial fibrillation in patients with cardiovascular disease, as well as the association between and with several cardiometabolic diseases, systemic inflammation markers, and the echocardiographic parameters associated with the presence and progression of atrial fibrillation.

## 2. Materials and Methods

### 2.1. Study Design

In a cross-sectional study design, we included 122 adult patients hospitalized for cardiovascular disease, randomly recruited in the cardiology department of the Clinical Rehabilitation Hospital in Cluj-Napoca, Romania. Patients on antimicrobial drugs at the time of admission and very sick patients were excluded, such as those with functional NYHA IV class, stage IV COPD, end-stage kidney disease with eGFR < 15 mL/min, patients with cancer and with uncontrolled systemic inflammatory diseases, and patients with type I diabetes mellitus. Atrial fibrillation and its disease progression phenotypes were classified according to the 2020 ESC Guidelines for the Management of Atrial Fibrillation. Moreover, 32 healthy subjects with no known disease were voluntarily recruited in order to establish a healthy control group. Patient history was obtained from the hospital records. In addition to the presence of AF, the patients were screened for seven comorbidities: arterial hypertension (HTN), atherosclerotic cardiovascular disease (ASCVD, defined as known chronic coronary syndrome, peripheral artery disease–carotid artery stenosis, aortic aneurysm, lower extremity disease, or previously known arterial revascularization procedures such as angioplasty or arterial bypass), heart failure (HF), the presence of type 2 diabetes mellitus (T2DM), chronic kidney disease (CKD), previous ischemic stroke and dyslipidemia. Additionally, data on medications at the time of admission were collected, specifically regarding the use of angiotensin-converting enzyme inhibitors (ACEi), angiotensin receptor blockers (ARBs), β-blockers, calcium channel blockers (CCBs), thiazide diuretics, mineralocorticoid receptor antagonists (MRAs), loop diuretics, statins, antiplatelet medications, oral anticoagulants (OACs), amiodarone, and type III antiarrhythmics. Data on echocardiography parameters such as left ventricular systolic function (Simpson disk summation method), diastolic function and left ventricular filling pressure estimation (the transmitral pulsed wave Doppler E/A ratio, tissue doppler-derived E/e’ ratio, left atrial antero-posterior diameter obtained in a parasternal short-axis view, and left atrial volume obtained in an apical 4-chamber view). The echography parameters were measured by more than one operator on a Philips Epiq 7 echography machine (Koninklijke Philips N.V., Amsterdam, The Netherlands). Patients were divided into three groups: healthy controls, patients with CVD and no AF, and patients with CVD and AF. The reason for admission was obtained from hospital records using the International Classification of Diseases 10th revision (ICD-10) code.

For the analysis of serum TMAO levels, blood was drawn from each patient on an empty stomach, at least 12 h after their last meal, in a Vacutainer containing a serum clot activator, after which it was stored in a freezer at −80 °C for further processing. After defrosting at room temperature, the TMAO serum levels were quantified using liquid chromatography tandem mass spectroscopy (LC-MS/MS) (Agilent 1100 LC-MSD-Trap-SL, Agilent Technologies, Santa Clara, CA, USA), using a method previously described [20]. Systemic inflammation was evaluated by measuring the serum interleukin-1B (IL-1β), interleukin 1A (IL-1α), and toll-like receptor (TLR) levels, using the ELISA method from commercially available kits (Elabscience, Houston, TX, USA).

The study was approved by the Ethics Board of the University of Medicine and Pharmacy “Iuliu Hatieganu” of Cluj-Napoca and by the Clinical Rehabilitation Hospital of Cluj-Napoca. All the patients agreed and signed written informed consent forms, in accordance with the Declaration of Helsinki.

### 2.2. Statistical Analysis

Data were analyzed using Prism GraphPad version 9.3 (GraphPad Software Inc., San Diego, CA, USA). Data were described as mean ± standard deviation (SD) for normally distributed variables and as median ± interquartile range (IQR) for nonnormally distributed variables. For the normality of the variables, the Shapiro–Wilk test was used. The comparison between normally distributed variables was evaluated using the Student’s *t* test and ANOVA for multivariate comparisons. For the comparison of non-normally distributed variables, Mann–Whitney and Kruskal–Wallis tests were used for multivariable comparison. Correlations were evaluated using Pearson’s correlation coefficient. Multiple linear regression was used to assess the association between variations in TMAO and the presence of cardiovascular disease and echography parameters.

## 3. Results

### 3.1. General Characteristics of the Population

**Figure 1:**
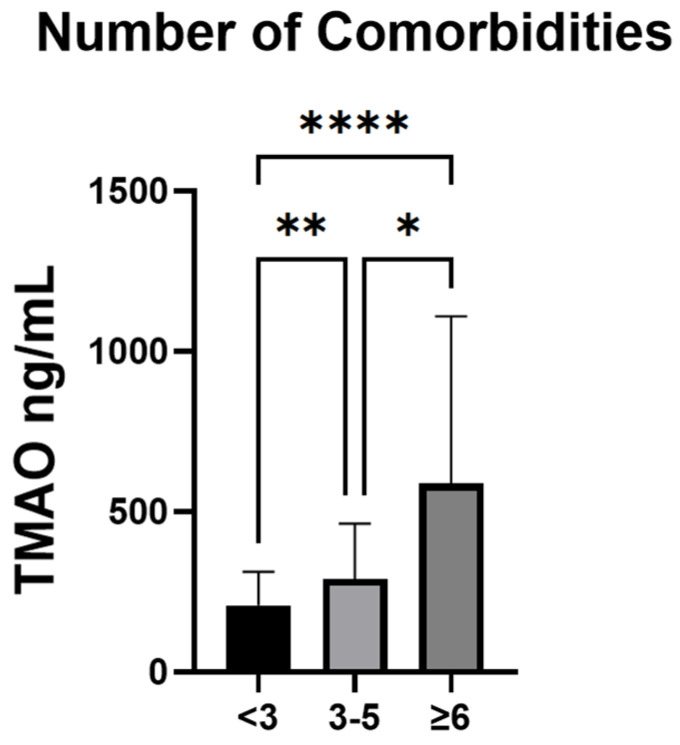
TMAO levels with regard to the number of comorbidities. Patients with more comorbidities had significantly higher mean levels of serum TMAO. Data expressed as mean ± SD, Kruskal–Wallis test with post hoc Dunn test. * *p* < 0.005, ** *p* < 0.01, **** *p* < 0.0001.

This study included 154 participants, with males constituting 63.12% and females 36.88%. The mean age was 65.63 (±1.05) years. Among these, 32 individuals (20.9%) were healthy controls without cardiovascular disease (CVD), 65 (42.1%) had CVD and atrial fibrillation (AF), and 57 (37%) had CVD without AF. In the AF group, the distribution was as follows: 22 had paroxysmal AF, 30 had persistent AF, and 13 had permanent AF. The main reason for admission was ASCVD in 36.06% of cases (for diagnosis and interventional therapy), AF in 29.50% of cases (mainly for ablation or cardioversion), decompensated HF in 15.57% of cases and other causes in 18.85% of cases (mainly other ablation procedures, device therapy, valvular disease). The average serum TMAO level in all groups was noted at 304.1 ng/mL (interquartile range [IQR] 163.3–358.5 ng/mL). The prevalence of studied conditions was significant: 76.23% had arterial hypertension, 49.18% had atherosclerotic cardiovascular disease, 27.5% had type 2 diabetes mellitus, 22.95% had chronic kidney disease at various stages, 15.57% had experienced a stroke, 84.43% had some form of heart failure, and 65.4% had dyslipidemia. Comparing patients with CVD with and without AF, those with AF had lower rates of ASCVD (*p* < 0.001) but higher instances of heart failure (*p* < 0.001), as detailed in Table 1. Additionally, patients with CVD and AF were more likely to be prescribed diuretics (loop diuretics and MRAs), β-blockers, OAC medication, and antiarrhythmics (such as amiodarone and type III antiarrhythmics). In contrast, patients with CVD but without AF were more likely to be on anti-platelet medication. Patients with more comorbidities had significantly higher serum TMAO values (208.74, IQR 123.8–281.9, with less than three comorbidities, 290.51, IQR 187.8–339.3 when between three and five comorbidities, 587.59, IQR 243.6–695.1, with six or more comorbidities, *p* < 0.001) (Figure 1). 

### 3.2. The Association Between Serum TMAO Levels and Atrial Fibrillation

**Figure 2:**
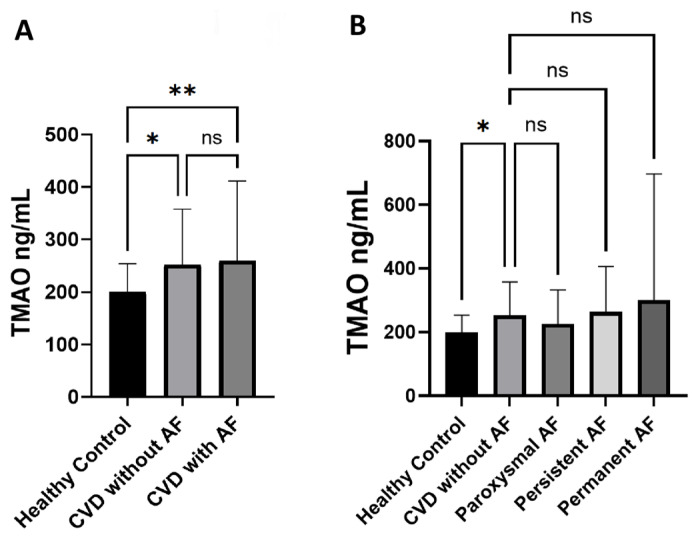
Serum TMAO levels in the study population. (**A**) Comparison between serum TMAO levels in healthy controls and patients with CVD with and without AF. (**B**) Comparison between serum TMAO levels in healthy controls, patients with CVD without AF, and clinical subtypes of AF. Data are expressed as median ± IQR. * *p* < 0.005, ** *p* < 0.01, ns—not significant, graphical comparison between CVD without AF, Kruskal–Wallis test, and post hoc Dunn’s test.

The serum TMAO levels were compared between healthy controls and CVD patients, stratified by the presence of AF (Figure 2). A significant difference was observed between AF patients and healthy controls (*p* = 0.03), but there was no difference between patients with CVD with or without AF (*p* = 0.57) (Figure 2A). We further analyzed the different clinical subtypes of AF, comparing them to both healthy controls and patients with CVD but without AF. The trend was similar, with significant differences observed between persistent AF (*p* = 0.01) and permanent AF (*p* = 0.004) compared to healthy controls. Interestingly, no significant difference existed between paroxysmal AF and healthy controls (*p* = 0.4) (Supplementary Figure S1). No differences were found between any of the AF subtypes and patients with CVD, without AF (*p* > 0.05 for all comparisons) (Figure 2B).

### 3.3. The Correlation of TMAO with Echocardiography Parameters

We assessed whether serum TMAO levels correlate with different echocardiographic parameters associated with AF in patients with CVD (with and without AF) (Table 2). We found a weak and significant positive correlation between serum TMAO levels and parameters of diastolic disfunction and LV filling pressures such as the E/A ratio (r = 0.270, CI 0.1169 to 0.4107, *p* = 0.001), anteroposterior LA diameter (r = 0.248, CI 0.07344 to 0.4078, *p* = 0.069), LA volume (r = 0.249, CI 0.07452 to 0.4087, *p* = 0.006), E/e’ ratio (r = 0.324, CI 0.1553 to 0.4745, *p* = <0.001) and an inverse weak correlation with LVEF (r = −0.330, CI −0.4639 to −0.1813, *p* = 0.0005).

### 3.4. Serum TMAO Levels in Associated Diseases

**Figure 3:**
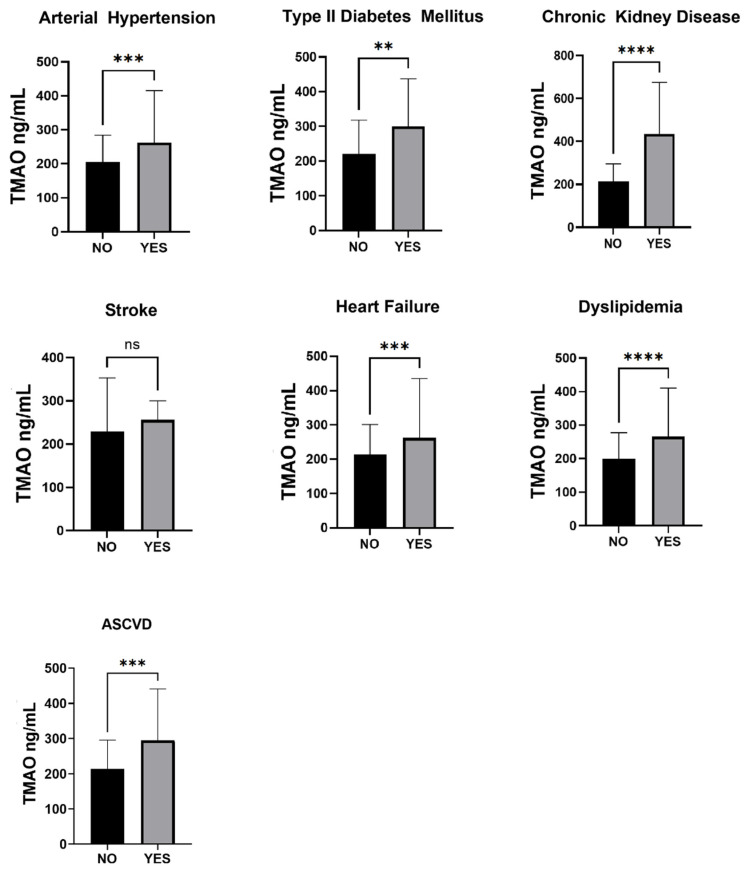
Serum TMAO levels in patients with common CVD and risk factors. Graphical representation of the difference in serum TMAO levels in patients with or without the specified disease. Data expressed as median ± IQR. ** *p* < 0.01, *** *p* < 0.001, **** *p* < 0.0001, ns—not significant. Mann–Whitney test.

We evaluated whether the TMAO levels were different in the context of the included common cardiovascular diseases and risk factors (Figure 3). There were significant differences in the serum TMAO levels in patients with and without HTN (*p* = 0.0009), T2DM (*p* = 0.0005), CDK (*p* < 0.0001), HF (*p* = 0.0009), dyslipidemia (*p* < 0.0001) and ASCVD (*p* = 0.0004), but no differences in stroke patients (*p* = 0.3). To further test the dependency of serum TMAO levels on the studied parameters and to assess for confounders, multiple linear regression analysis was used (Supplementary Table S1). When adjusting for confounders, serum levels were only dependent on the presence of ASCVD (*p* = 0.043) and CKD (*p* < 0.001).

### 3.5. Systemic Inflammatory Markers in AF Patients

**Figure 4:**
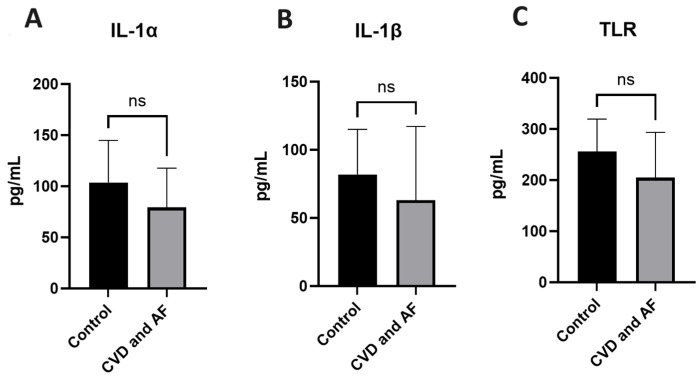
Serum inflammatory markers in AF patients. Graphical representation of serum inflammatory markers IL-1α (**A**), IL-1β (**B**) and TLR (**C**), in AF patients compared to the healthy control group. Data expressed as median ± IQR. Mann–Whitney test. ns—not significant.

We further studied the association between AF and inflammation by comparing the serum levels of the systemic inflammation markers IL-1α, IL-1β, and TLR (Figure 4) between patients with CVD and AF and the healthy control group. We found no difference in the mean levels of IL-1α (*p* = 0.31), IL-1β (*p* = 0.21), and TLR (*p* = 0.25) when compared to the Healthy Control group. Moreover, there was no significant correlation between TMAO and the studied inflammatory markers: r = 0.002 (CI −0.2399 to 0.2856) with IL-1 α, r = 0.11 (CI −0.2390 to 0.4496) with IL IL-1β, and r = 0.12 (CI −0.1459 to 0.3727) with TLR.

## 4. Discussion

The relationship between serum TMAO levels and the occurrence of AF in patients with known cardiovascular disease who required evaluation in an inpatient setting in a cardiovascular rehabilitation ward was analyzed. Furthermore, we assessed the association of TMAO with common cardiovascular diseases and the risk factors associated with AF, such as atherosclerotic cardiovascular disease, heart failure, stroke, arterial hypertension, type 2 diabetes mellitus, and chronic kidney disease, and with echography parameters related to the occurrence and progression of AF. A cross-sectional study design was used to obtain a broad overview of these relationships within a specific timeframe and study the TMAO levels in individuals with varying degrees of cardiovascular disease and AF phenotypes, in a real-world clinical scenario.

In this pilot, exploratory study, serum TMAO does not appear to be associated with the incidence of AF in any of its clinical forms compared to patients with known CVD, but its incidence is higher compared to healthy controls. The results were consistent with the results of some other studies. Thus, in a comparative study, Büttner et al. included 45 patients undergoing catheter ablation for AF, comparing serum TMAO levels to 20 patients with CVD but without AF. They found no significant differences in the serum TMAO levels between AF and non-AF patients (3.5 µM [2.51–4.53] versus 3.62 µM [2.49–5.46], *p* = 0.629) and no association between TMAO and AF progression phenotypes (*p* = 0.58) [17]. In a Norwegian study of 3797 patients with confirmed and suspected angina pectoris from the WECAC and HUSK cohorts, Svingen et al. noticed that TMAO was associated with incident AF after a follow-up period of more than 7 years, independent of the traditional risk factors linked with AF or with dietary choline intake [21]. In a retrospective clinical study by Nguyen et al. on a series of patients included in the AF-RISK study with paroxysmal or persistent AF, higher plasma TMAO levels were significantly associated with a more progressed form of AF (5.65 [4.7–9.6] *m*/*z* for persistent AF versus 4.31 [3.2–6.2] *m*/*z* for paroxysmal AF, *p* < 0.05). In addition, the odds of having persistent AF increased with every 0.44 per unit of TMAO *m*/*z* increase [16]. In an interesting experimental study on the effect of AF inducibility in a canine experimental model, Yu et al. directly injected TMAO into major para-cardiac autonomous ganglionated plexi and induced atrial electrophysiological changes related to AF progression, significantly shortened the atrial effective refractory period, and increased the ganglionated plexi neural activity and function. Furthermore, TMAO induced the activation of the pro-inflammatory p65 NF-κB signaling pathway, which may contribute to the progression of the disease [14]. Finally, in a meta-analysis of the effect of TMAO on the incidence of AF, Yang et al. found that TMAO was indeed associated with AF in a dose-dependent manner, increasing the risk of AF by 6% per every 1 µmol/L increment, 32% per every 5 µmol/L increment, and 73% per every 10 µmol/L increment in TMAO levels [15]. However, studies evaluating the relationship between AF and TMAO are still few, and their results tend to show a positive, albeit weak, correlation. In our study, we did not find a correlation between TMAO and AF in stable patients with chronic CVD. In general, higher levels of TMAO appear to be associated with patients with more comorbidities. The selection of the patients, the number of comorbidities and confounder adjustment could explain the differences between the above-mentioned findings. The obtained results were divergent probably also due to the inclusion criteria in the study or due to the small groups of patients with AF and CVD.

When comparing the serum TMAO levels in each individual disease or risk factor, we found that TMAO was higher in each of the studied diseases, except for stroke. However, in the multivariate analysis, TMAO was associated only with ASCVD and CKD. The association with ASCVD is well known and in concordance with most clinical and experimental studies [22]. The association with CKD is also well documented, partly because TMAO is eliminated by the kidney, but studies also suggest that it plays a direct role in the development and progression of kidney disease [23]. The lack of an association with stroke is inconsistent with most studies, in which TMAO levels were found to be an independent predictor of stroke [24], vascular brain lesions [25], or thrombus formation [26] in AF patients. TMAO seems to be significantly correlated with echography parameters related to diastolic dysfunction and atrial size, such as the mitral pulsed-wave doppler E/A ratio, LA size and volume, and tissue doppler E/e’ ratio, and inversely correlated with LEVF, although the correlations are weak. In a study evaluating the effect of TMAO on chronic HF patients, blood TMAO levels were not correlated with the E/A ratio (r = 0.07) or with LVEF (r = −0.10), but were correlated with the invasively measured pulmonary capillary wedge pressure (r = 0.36, *p* < 0.01), an accurate marker of left atrial pressure and left ventricular filling pressure [27]. It is unclear if TMAO itself can induce echocardiographic alterations, or if the findings can be explained by the presence of multiple comorbidities or more advanced disease in these patients. Once again, few studies have evaluated the relationship between serum IL-1β and AF [28,29], and found no association, in concordance with our results. To our knowledge, no other studies have evaluated the serum levels of IL-1α and TLR in AF patients. These results were somewhat unexpected, given the fact that oxidative stress, DNA damage, and inflammation are core pathogenetic mechanisms in AF [30,31]. Furthermore, inflammatory cytokines are known to be elevated in various CVD patients, so we would have expected the markers studied to be higher, especially given the relatively high number of comorbidities in the studied population. This is particularly considering that, in our experimental studies, a strong correlation was observed between TMAO levels and inflammation, oxidative stress, endothelial dysfunction and incipient atherosclerosis. It is likely that larger studies, performed on more patients with FA and evaluating other more specific inflammation parameters, would lead to more accurate results regarding the involvement of inflammation in the pathogenesis and evolution of AF.

We acknowledge several limitations to this study. Firstly, the study is descriptive in nature and cannot establish causality between TMAO and the variables studied. Furthermore, the study sample was relatively small, mainly for generating a hypothesis, so robust conclusions cannot be drawn. Additionally, comparisons between healthy controls and patients with AF but no CVD could not be made due to the low number of patients with AF without CVD included in the study. Regarding the impact of food on serum TMAO levels, blood was collected during fasting, more than 12 h after the last meal, and while the half-life of blood is relatively short, approximately 4 h, one cannot account for the whole variation in individual pharmacokinetics. While genetic predisposition influences TMAO levels, diet remains a significant modifiable determinant, as dietary intake, particularly of choline-rich foods like red meat, eggs, and dairy, strongly affects serum TMAO concentrations through the gut microbiota’s metabolism of dietary precursors [32]. In addition, it is possible that patients suffered from subclinical disease or other diseases that may have influenced the serum TMAO levels. Echography parameters were not measured double blind, so the values are subjected to inter-individual variation.

## 5. Conclusions

In conclusion, serum TMAO levels are not associated with the presence of atrial fibrillation and disease progression phenotypes in patients with cardiovascular disease or with systemic inflammation, but are associated with more sick patients, atherosclerotic cardiovascular disease and chronic kidney disease.

## Figures and Tables

**Table 1:** Study population characteristics. Data expressed as mean ± SD for the age variable. Data expressed as percentage for patients with known CVD with AF and without AF for the rest of the variables. * *p* < 0.05, *t*-test for the age variable and Chi-squared test for the rest of the variables.

	CVD Without AF *n* = 57	CVD with AF *n* = 65	*p*-Value
**Age**	67.61 ± 1.31	64.61 ± 1.66	0.072
**Sex**			
Male	59.65	67.69	0.351
Female	40.35	32.31	0.083
**Comorbidities**			
HTN	68.42	83.08	0.068
ASCVD	63.16	36.92	<0.001 *
T2DM	31.58	23.08	0.072
CKD	17.54	27.69	0.182
Dyslipidemia	85.91	83.08	0.801
HF	45.61	75.38	<0.001 *
Stroke	10.53	20.12	0.149
**Medication**			
ACEi/ARB	84.21	86.15	0.764
β-blocker	75.43	92.30	0.012 *
CCB	29.82	23.07	0.395
Thiazide	35.08	24.61	0.204
Loop diuretic	19.29	47.69	0.015 *
MRA	8.77	35.38	<0.001 *
Digoxin	0	15.38	0.002 *
Statins	80.70	84.61	0.560
Anti-platelet agents	63.15	10.76	<0.001 *
OAC	1.75	96.92	<0.001 *
Amiodarone	0	43.07	<0.001 *
Type III antiarrhythmics	0	18.46	<0.001 *

**Table 2:** The correlation between TMAO and echocardiographic features. Values expressed as Pearson’s correlation coefficient (r). * *p* < 0.05.

	Correlation Coefficient	*p* Value
**E/A RATIO**	0.270	0.001 *
**LA AP DIAMETER**	0.248	0.006 *
**LA VOLUME**	0.249	0.006 *
**E/e’ RATIO**	0.324	<0.001 *
**LVEF**	−0.330	<0.005 *

## Data Availability

The corresponding author can provide the datasets upon reasonable request.

## Supplementary Materials

The following supporting information can be downloaded at: https://www.mdpi.com/article/10.3390/jox15010028/s1, Figure S1: Comparison of serum TMAO in healthy patients, patients with CVD without AF and clinical AF subtypes; Table S1: Results of multiple linear regression.
