# Supplementary material for: The Impact of Trimethylamine N-Oxide on Atrial Fibrillation Presence in Patients with Cardiovascular Disease
Source: J Xenobiot. 2025 Feb 7;15(1):28. doi: 10.3390/jox15010028 (PMC11856497; doi:10.3390/jox15010028)
Supplement: Supplementary file 1 [file jox-15-00028-s001.zip › Supplementary Table S1.pdf]

Table S1. Results of the multiple linear regression analysis for the assessment of the dependency of serum TMAO levels on the included diseases.

Table Analyzed	Multiple variable analysis
Dependent variable	TMAO
Regression type	Least squares

**Model**					
**Analysis of Variance**	**SS**	**DF**	**MS**	**F (DFn, DFd)**	**P value**
**Regression**	3132779	8	391597	F (8, 113) = 6.463	P<0.0001
**AF**	3961	1	3961	F (1, 113) = 0.06536	P=0.7987
**HTN**	22994	1	22994	F (1, 113) = 0.3795	P=0.5391
**ASCVD**	251917	1	251917	F (1, 113) = 4.157	P=0.0438
**CKD**	1293993	1	1293993	F (1, 113) = 21.35	P<0.0001
**DM**	2252	1	2252	F (1, 113) = 0.03717	P=0.8475
**Stroke**	55838	1	55838	F (1, 113) = 0.9215	P=0.3391
**HF**	33071	1	33071	F (1, 113) = 0.5458	P=0.4616
**Dyslipidemia**	1109	1	1109	F (1, 113) = 0.01830	P=0.8926
**Residual**	6847261	113	60595		
**Total**	9980040	121			

**Parameter estimates**	**Variable**	**Estimate**	**Standard error**	**95% CI (asymptotic)**	**|t|**	**P value**	**P value summary**
β0	Intercept	154.3	64.38	26.73 to 281.8	2.396	0.0182	*
β1	AF[da]	13.40	52.41	-90.44 to 117.2	0.2557	0.7987	ns
β2	HTN[da]	36.51	59.26	-80.90 to 153.9	0.6160	0.5391	ns
β3	ASCVD[da]	115.3	56.54	3.267 to 227.3	2.039	0.0438	*
β4	CKD[da]	280.7	60.74	160.3 to 401.0	4.621	<0.000 ****	
β5	DM[da]	-10.63	55.14	-119.9 to 98.61	0.1928	0.8475	ns
β6	Stroke[da]	-63.32	65.96	-194.0 to 67.36	0.9599	0.3391	ns
β7	HF[da]	40.65	55.02	-68.36 to 149.6	0.7388	0.4616	ns
β8	Dyslipidemia[da]	10.38	76.75	-141.7 to 162.4	0.1353	0.8926	ns

**Goodness of Fit**	
Degrees of Freedom	113
R squared	0.3139

**Multicollinearity**	**Variable**	**VIF**	**R2 with other variables**
β0	Intercept		
β1	AF[da]	1.377	0.2737
β2	HTN[da]	1.340	0.2538
β3	ASCVD[da]	1.605	0.3770
β4	CKD[da]	1.313	0.2387
β5	DM[da]	1.208	0.1721
β6	Stroke[da]	1.152	0.1318
β7	HF[da]	1.419	0.2952
β8	Dyslipidemia[da]	1.559	0.3588

**Normality of Residuals**	**Statistics**	**P value**	**Passed normality test (alpha=0.05)?**	**P value summary**
Anderson-Darling (A2*)	7.239	<0.0001	No	****
D'Agostino-Pearson omnibus (K2)	132.2	<0.0001	No	****
Shapiro-Wilk (W)	0.7088	<0.0001	No	****
Kolmogorov-Smirnov (distance)	0.1812	<0.0001	No	****
