# Supplementary figures and images for: The Impact of Trimethylamine N-Oxide on Atrial Fibrillation Presence in Patients with Cardiovascular Disease
Source: J Xenobiot. 2025 Feb 7;15(1):28. doi: 10.3390/jox15010028 (PMC11856497; doi:10.3390/jox15010028)

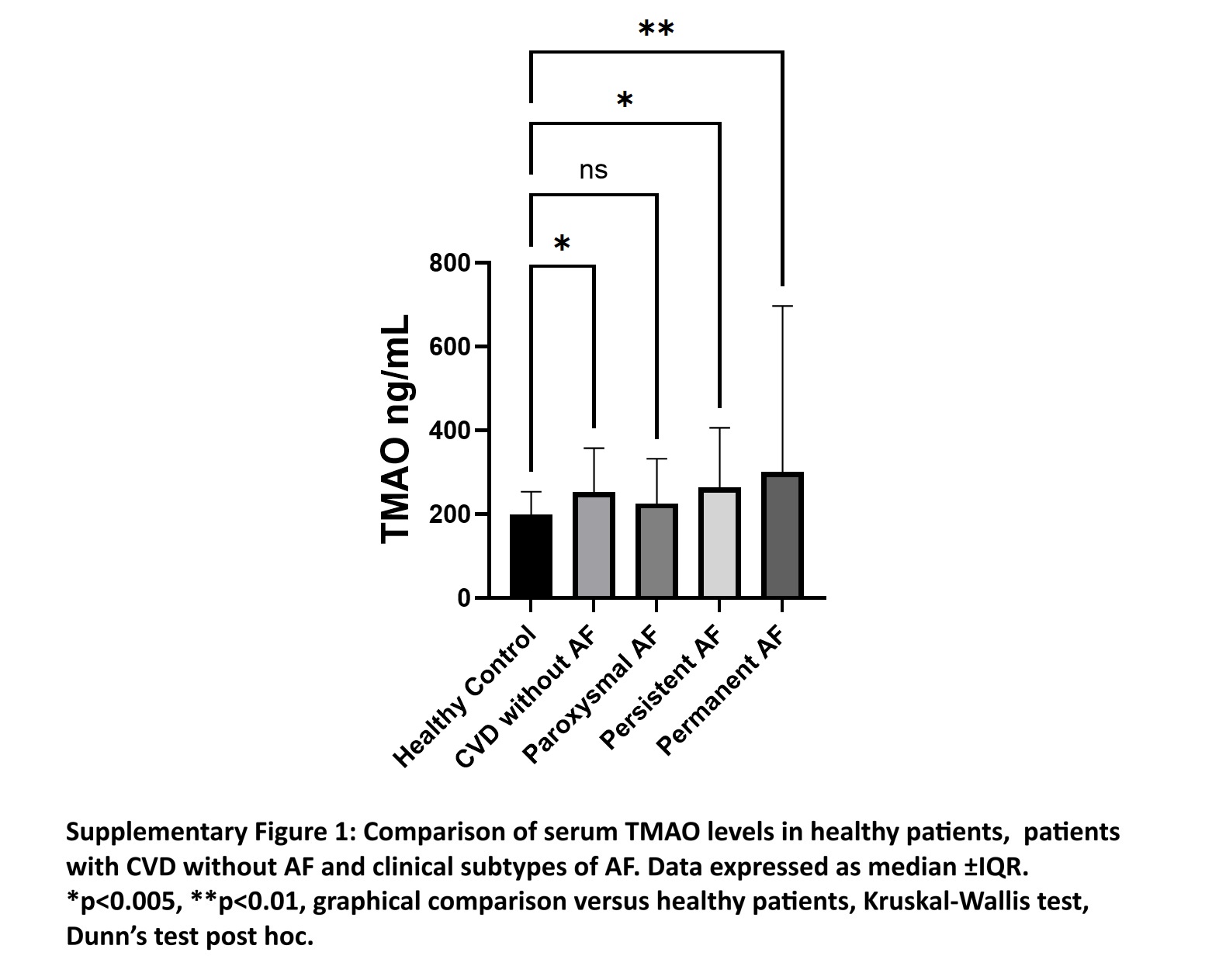

Supplement: Supplementary file 1 [file jox-15-00028-s001.zip › Supplementary figure S1.jpg]
